# Supplementary material for: Iterative improvement in the automatic modular design of robot swarms
Source: PeerJ Comput Sci. 2020 Dec 7;6:e322. doi: 10.7717/peerj-cs.322 (PMC7924708; doi:10.7717/peerj-cs.322)
Supplement: Supplemental Information 3 [file peerj-cs-06-322-s003.zip › argos3/doc/api/standalone/a00400_source.html]

ARGoS: core/utility/plugins/factory\_impl.h Source File


- Main Page
- Related Pages
- Namespaces
- Classes
- Files

- File List
- File Members

# core/utility/plugins/factory\_impl.h

Go to the documentation of this file.

```
00001 
00011 #ifndef FACTORY_IMPL_H
00012 #define FACTORY_IMPL_H
00013 
00014 /****************************************/
00015 /****************************************/
00016 
00017 template<typename TYPE>
00018 typename CFactory<TYPE>::TTypeMap& CFactory<TYPE>::GetTypeMap() {
00019    static typename CFactory<TYPE>::TTypeMap tTypeMap;
00020    return tTypeMap;
00021 }
00022 
00023 /****************************************/
00024 /****************************************/
00025 
00026 template<typename TYPE>
00027 void CFactory<TYPE>::Register(const std::string& str_label,
00028                               const std::string& str_author,
00029                               const std::string& str_version,
00030                               const std::string& str_brief_desc,
00031                               const std::string& str_long_desc,
00032                               const std::string& str_status,
00033                               TCreator* pc_creator) {
00034    typename CFactory<TYPE>::STypeInfo* psTypeInfo = new typename CFactory<TYPE>::STypeInfo;
00035    psTypeInfo->Author = str_author;
00036    psTypeInfo->Version = str_version;
00037    psTypeInfo->BriefDescription = str_brief_desc;
00038    psTypeInfo->LongDescription = str_long_desc;
00039    psTypeInfo->Status = str_status;
00040    psTypeInfo->Creator = pc_creator;
00041    GetTypeMap()[str_label] = psTypeInfo;
00042 }
00043 
00044 /****************************************/
00045 /****************************************/
00046 
00047 template<typename TYPE>
00048 TYPE* CFactory<TYPE>::New(const std::string& str_label) {
00049    typename TTypeMap::iterator it = GetTypeMap().find(str_label);
00050    if(it != GetTypeMap().end()) {
00051       return it->second->Creator();
00052    }
00053    else {
00054       THROW_ARGOSEXCEPTION("Symbol \"" << str_label << "\" not found");
00055    }
00056 }
00057 
00058 /****************************************/
00059 /****************************************/
00060 
00061 template<typename TYPE>
00062 bool CFactory<TYPE>::Exists(const std::string& str_label) {
00063    typename TTypeMap::iterator it = GetTypeMap().find(str_label);
00064    return(it != GetTypeMap().end());
00065 }
00066 
00067 /****************************************/
00068 /****************************************/
00069 
00070 template<typename TYPE>
00071 void CFactory<TYPE>::Print(std::ostream& c_os) {
00072    typename TTypeMap::iterator it;
00073    c_os << "Symbols:" << std::endl;
00074    for(it = GetTypeMap().begin();
00075        it != GetTypeMap().end();
00076        ++it) {
00077       c_os << it->first << " (" << it->second->BriefDescription << ")" << std::endl;
00078    }
00079 }
00080 
00081 /****************************************/
00082 /****************************************/
00083 
00084 template<typename TYPE>
00085 void CFactory<TYPE>::Destroy() {
00086    typename TTypeMap::iterator it;
00087    for(it = GetTypeMap().begin();
00088        it != GetTypeMap().end();
00089        ++it) {
00090       delete it->second;
00091    }
00092    GetTypeMap().clear();
00093 }
00094 
00095 /****************************************/
00096 /****************************************/
00097 
00098 #endif
```

---

Generated on 10 Jul 2018 for ARGoS by 
 1.6.1
